# Ensemble machine learning modeling for the prediction of artemisinin resistance in malaria

**DOI:** 10.12688/f1000research.21539.2

**Published:** 2020-02-04

**Authors:** Colby T. Ford, Daniel Janies

**Affiliations:** 1 Department of Bioinformatics and Genomics, University of North Carolina at Charlotte, Charlotte, North Carolina, 28223, USA; 2 School of Data Science, University of North Carolina at Charlotte, Charlotte, North Carolina, 28223, USA

**Keywords:** malaria, Plasmodium falciparum, machine learning, parallel computing, Apache Spark, big data, artemisinin, bioinformatics, DREAM Competition

## Abstract

Resistance in malaria is a growing concern affecting many areas of Sub-Saharan Africa and Southeast Asia. Since the emergence of artemisinin resistance in the late 2000s in Cambodia, research into the underlying mechanisms has been underway.

The 2019 Malaria Challenge posited the task of developing computational models that address important problems in advancing the fight against malaria. The first goal was to accurately predict artemisinin drug resistance levels of *Plasmodium falciparum* isolates, as quantified by the IC _50_. The second goal was to predict the parasite clearance rate of malaria parasite isolates based on *in vitro* transcriptional profiles.

In this work, we develop machine learning models using novel methods for transforming isolate data and handling the tens of thousands of variables that result from these data transformation exercises. This is demonstrated by using massively parallel processing of the data vectorization for use in scalable machine learning. In addition, we show the utility of ensemble machine learning modeling for highly effective predictions of both goals of this challenge. This is demonstrated by the use of multiple machine learning algorithms combined with various scaling and normalization preprocessing steps. Then, using a voting ensemble, multiple models are combined to generate a final model prediction.

## Introduction

Malaria is a serious disease caused by parasites belonging to the genus *Plasmodium* which are transmitted by *Anopheles* mosquitoes in the genus. The World Health Organization (WHO) reports that there were 219 million cases of malaria in 2017 across 87 countries ^1^. *Plasmodium falciparum* poses one of greatest health threats in Southeast Asia, being responsible for 62.8% of malaria cases in the region in 2017 ^1^.

Artemisinin-based therapies are among the best treatment options for malaria caused by *P. falciparum* ^2^. However, emergence of artemisinin resistance in Thailand and Cambodia in 2007 has been cause for research ^3^. While there are polymorphisms in the kelch domain–carrying protein K13 in *P. falciparum* that are known to be associated with artemisinin resistance, the underlying molecular mechanism that confers resistance remains unknown ^4^. The established pharmacodynamics benchmark for *P. falciparum* sensitivity to artemisinin-based therapy is the parasite clearance rate ^5, 6^. Resistance to artemisinin-based therapy is considered to be present with a parasite clearance rate greater than five hours ^7^. By understanding the genetic factors that affect resistance in malaria, targeted development can occur in an effort to abate further resistance or infections of resistant strains.

## Prediction of artemisinin IC _50_

First, we created a machine learning model to predict the IC _50_ of malaria parasites based on transcription profiles of experimentally-tested isolates. IC _50_, also known as the half maximal inhibitory concentration, is the drug concentration at which 50% of parasites die. This value indicates a population of parasites’ ability to withstand various doses of anti-malarial drugs, such as artemisinin.

### Methods

Training data was obtained from the 2019 DREAM Malaria Challenge ^8, 9^. The training data consists of gene expression data of 5,540 genes of 30 isolates from the malaria parasite, *Plasmodium falciparum*. For each malaria parasite isolate, transcription data was collected at two time points [6 hours post invasion (hpi) and 24 hpi], with and without treatment of dihydroartemisinin (the metabolically active form of artemisinin), each with a biological replicate. This yields a total of at eight data points for each isolate. The initial form of the training dataset contains 272 rows and 5,546 columns, as shown in Table 1.

**Table 1.** Initial IC _50_ model training data format.

Sample_Name	Isolate	Timepoint	Treatment	BioRep	Gene _1_	…	Gene _5540_	DHA_IC50
isolate_01.24HR.DHA.BRep1	isolate_01	24HR	DHA	BRep1	0.008286	…	-2.48653	2.177
isolate_01.24HR.DHA.BRep2	isolate_01	24HR	DHA	BRep2	-0.87203	…	-1.79457	2.177
isolate_01.24HR.UT.BRep1	isolate_01	24HR	UT	BRep1	0.03948	…	-2.49517	2.177
isolate_01.24HR.UT.BRep2	isolate_01	24HR	UT	BRep2	0.125177	…	-1.73531	2.177
isolate_01.6HR.DHA.BRep1	isolate_01	6HR	DHA	BRep1	1.354956	…	-0.82169	2.177
isolate_01.6HR.DHA.BRep2	isolate_01	6HR	DHA	BRep2	-0.21807	…	-1.61839	2.177
isolate_01.6HR.UT.BRep1	isolate_01	6HR	UT	BRep1	1.31135	…	-2.62262	2.177
isolate_01.6HR.UT.BRep2	isolate_01	6HR	UT	BRep2	0.997722	…	-2.24719	2.177
…	…	…	…	…	…	…	…	…
isolate_30.6HR.UT.BRep2	isolate_30	6HR	UT	BRep2	-0.26639	…	-1.72273	1.363

The transcription data was collected as described in Table 2. The transcription data set consists of 92 non-coding RNAs (denoted by gene IDs that begins with ’MAL’), while the rest are protein coding genes (denoted by gene IDs that start with ’PF3D7’). The feature to predict is *DHA*_ *IC*50.

**Table 2.** IC _50_ training data information. (Adapted from Turnbull *et al*., (2017) PLoS One ^11^).

	Training Set
Array	Bozdech
Platform	Printed
Plexes	1
Unique Probes	10159
Range of Probes per Exon	N/A
Average Probes per Gene	2
Genes Represented	5363
Transcript Isoform Profiling	No
ncRNAs	No
Channel Detection Method	Two Color
Scanner	PowerScanner
Data Extraction	GenePix Pro

### Data preparation

We used Apache Spark ^10^ to pivot the dataset such that each isolate was its own row and each of the transcription values for each gene and attributes (i.e. timepoint, treatment, biological replicate) combination was its own column. This exercise transformed the training dataset from 272 rows and 5,546 columns to 30 rows and 44,343 columns, as shown in Table 3. We completed this pivot by slicing the data by each of the eight combinations of timepoint, treatment, and biological replicate, dynamically renaming the variables (genes) for each slice, and then joining all eight slices back together.

**Table 3.** Post-transformation format of the IC _50_ model training data.

Isolate	DHA_IC50	hr24_trDHA_br1_Gene _1_	hr24_trDHA_br2_Gene _1_	…	hr6_trUT_br2_Gene _5540_
isolate_01	2.177	0.008286	-0.87203	…	-2.24719
…	…	…	…	…	…
isolate_30	1.363	0.195032	0.031504	…	-1.72273

Example code shown below in the section labeled code 1. By using the massively parallel architecture of Spark, this transformation can be completed in a minimal amount of time on a relatively small cluster environment (e.g., <10 minutes using a 8-worker/36-core cluster with PySpark on Apache Spark 2.4.3).


 1 ## Separate Dependent Variable                                               
 2 y = train.select(col("Isolate"),                                             
 3                  col("DHA_IC50")) \                                          
 4          .distinct()                                                         
 5                                                                              
 6 ##  Create Slice [Timepoint: 24HR, Treatment: DHA, BioRep: BRep1]            
 7 hr24_trDHA_br1 = train.drop("Sample_Name","DHA_IC50") \                      
 8                       .filter((col("Timepoint") == "24HR") &                 
 9                               (col("Treatment") == "DHA") &                  
10                               (col("BioRep") == "BRep1"))                                                                
11 ## Rename Columns                                                            
12 column_list = hr24_trDHA_br1.columns                                         
13 prefix = "hr24_trDHA_br1_"                                                   
14 new_column_list = [prefix + s if s != "Isolate" else s for s in column_list] 
15                                                                              
16 column_mapping = [[o, n] for o, n in zip(column_list, new_column_list)]      
17                                                                              
18 hr24_trDHA_br1 = hr24_trDHA_br1.select(list(map(lambda old, new: col(old) \  
19                                .alias(new),*zip(*column_mapping))))          
20                                                                              
21 ## Join Slices Together                                                      
22 data = y.join(hr24_trDHA_br1, "Isolate", how='left') \                       
23         .join(hr24_trDHA_br2, "Isolate", how='left') \                       
24         .join(hr24_trUT_br1, "Isolate", how='left') \                        
25         .join(hr24_trUT_br2, "Isolate", how='left') \                        
26         .join(hr6_trDHA_br1, "Isolate", how='left') \                        
27         .join(hr6_trDHA_br2, "Isolate", how='left') \                        
28         .join(hr6_trUT_br1, "Isolate", how='left') \                         
29         .join(hr6_trUT_br2, "Isolate", how='left')                           


Lastly, the dataset is then vectorized using the Spark VectorAssembler, and converted into a Numpy ^12^-compatible array. Example code shown below in Code 1. Vectorization allows for highly scalable parallelization of the machine learning modeling in the next step.


 1 ## Transform Data using VectorAssembler                                          
 2 assemblerInputs = numericalColumns                                                 
 3 assembler = VectorAssembler(inputCols = assemblerInputs, outputCol="features") \ 
 4             .setHandleInvalid("keep")                                            
 5 stages += [assembler]                                                            
 6                                                                                  
 7 prepPipeline = Pipeline().setStages(stages)                                      
 8 pipelineModel = prepPipeline.fit(data)                                           
 9 vectordata = pipelineModel.transform(data) \                                     
10                           .select(col("DHA_IC50"), col("features")) \            
11                           .withColumnRenamed("DHA_IC50","label")                 
12                                                                                  
13 ## Convert to Numpy Array                                                        
14 import numpy as np                                                               
15 pddata = vectordata.toPandas()                                                   
16 seriesdata = pddata['features'].apply(lambda x : np.array(x.toArray())) \        
17              .as_matrix().reshape(-1,1)                                          
18 X_train = np.apply_along_axis(lambda x : x[0], 1, seriesdata)                    
19 y_train = pddata['label'].values.reshape(-1,1).ravel()                           
20                                                                                  
21 ## Example Output (X_train) After Vectorization                                  
22 array([[-0.62161893, -0.60860881, -1.11331369, ..., -1.457377 ,                  
23         -3.292903  , -1.869169  ],                                               
24        [-0.55719008, -2.41660489, -1.39244109, ..., -1.770098 ,                  
25         -3.698841  , -1.740082  ],                                               
26        ...,                                                                      
27        [-0.17072536, -2.32828532, -1.08406554, ..., -1.402658 ,                  
28          -5.314896 , -1.328886  ],                                               
29        [-0.1923732 , -1.88763881, -1.23867258, ..., -1.971246 ,                  
30          -3.567355 , -1.904116  ]])                                              


### Machine learning

We used the Microsoft Azure Machine Learning Service ^13^ as the tracking platform for retaining model performance metrics as the various models were generated. For this use case, 498 machine learning models were trained using various scaling techniques and algorithms. We then created two ensemble models of the individual models using Stack Ensemble and Voting ensemble methods. Scaling and normalization methods are shown in Table 14.

The Microsoft AutoML package ^14^ allows for the parallel creation and testing of various models, fitting based on a primary metric. For this use case, models were trained using Decision Tree, Elastic Net, Extreme Random Tree, Gradient Boosting, Lasso Lars, LightGBM, RandomForest, and Stochastic Gradient Decent algorithms along with various scaling methods from Maximum Absolute Scaler, Min/Max Scaler, Principal Component Analysis, Robust Scaler, Sparse Normalizer, Standard Scale Wrapper, Truncated Singular Value Decomposition Wrapper (as defined in Table 14). All of the machine learning algorithms are from the *scikit-learn* package ^15^ except for LightGBM, which is from the *LightGBM* package ^16^. The settings for the model sweep are defined in Table 4. The ‘Preprocess Data?’ parameter enables the scaling and imputation of the features in the data.

**Table 4.** Model search parameter setting for the IC _50_ model search.

Parameter	Value
Task	Regression
Number of Iterations	500
Iteration Timeout (minutes)	20
Max Cores per Iteration	7
Primary Metric	Normalized Root Mean Squared Error
Preprocess Data?	True
k-Fold Cross-Validations	20 folds

Once the 498 individual models were trained, two ensemble models (voting ensemble and stack ensemble) were then created and tested. The voting ensemble method makes a prediction based on the weighted average of the previous models’ predicted regression outputs whereas the stacking ensemble method combines the previous models and trains a meta-model using the elastic net algorithm based on the output from the previous models. The model selection method used was the Caruana ensemble selection algorithm ^17^.

### Results

**Figure 1.**
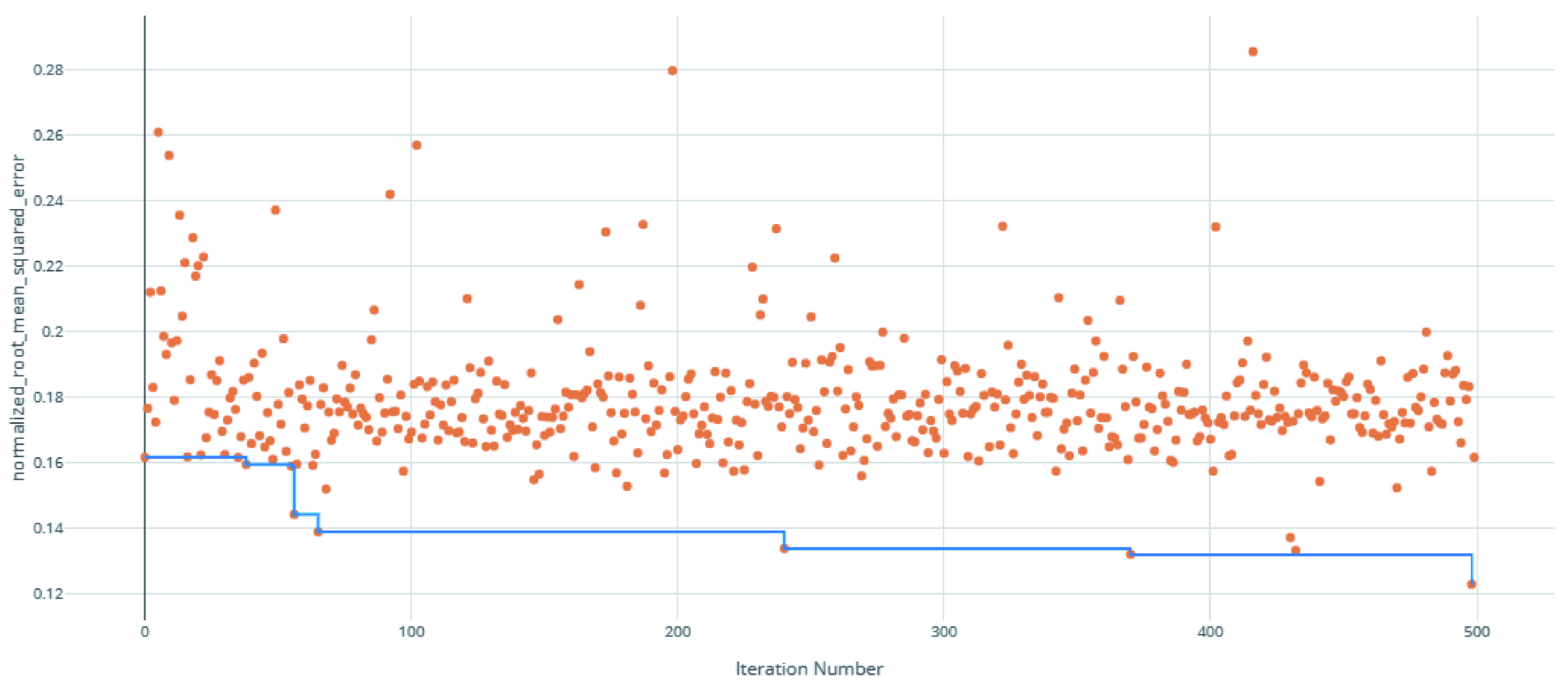
Root Mean Squared Error (RMSE) by iteration of the IC _50_ model search. Each orange dot is an iteration with the blue line representing the minimum RMSE up to that iteration.

**Figure 2.**
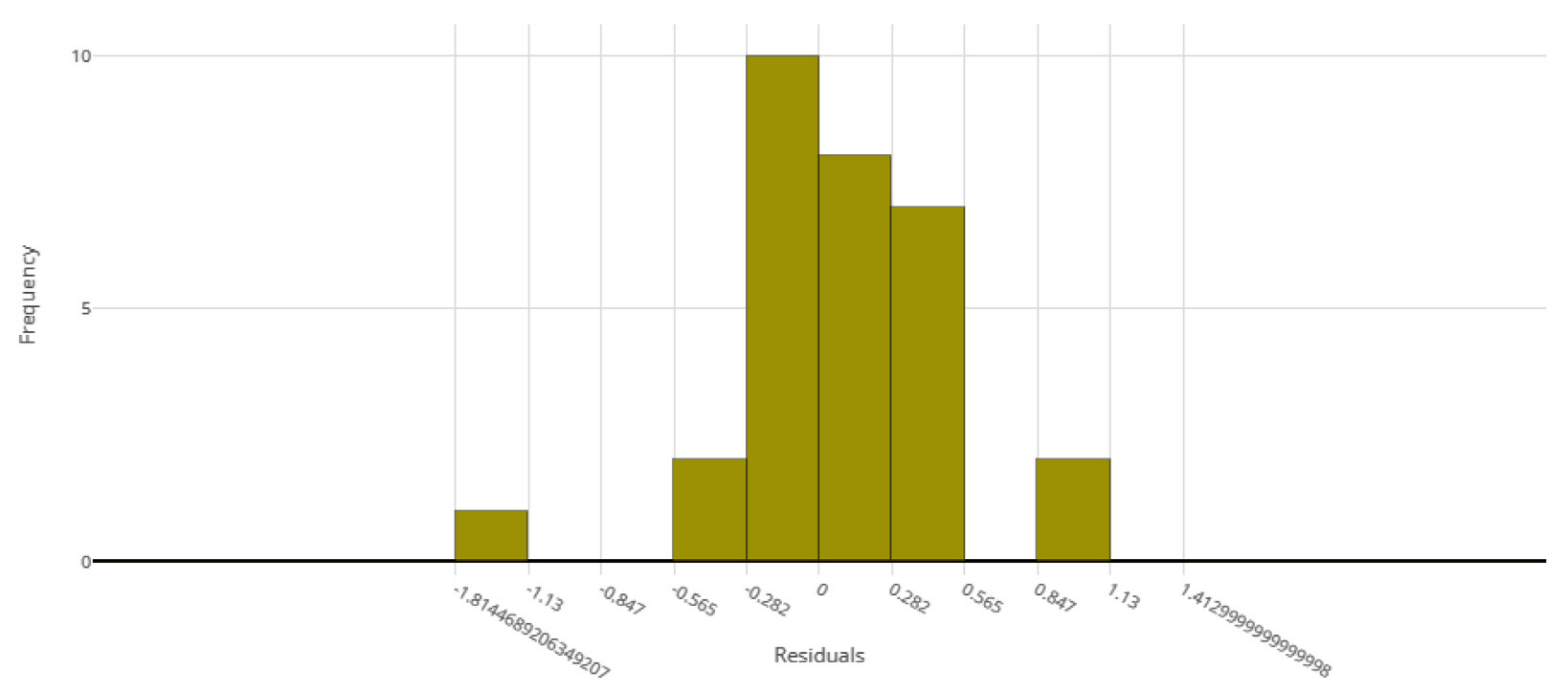
Model residuals of the final IC _50_ ensemble model.

The voting ensemble model (using soft voting) was selected as the best model, having the lowest normalized Root Mean Squared Error (RMSE), as shown in Table 5. The top 10 models trained are reported in Table 6. Having a normalized RMSE of only 0.1228 and a Mean Absolute Percentage Error (MAPE) of 24.27%, this model is expected to accurately predict IC _50_ in malaria isolates. See Figure 1 for a visualization of the experiment runs and Figure 2 for the distribution of residuals on the best model.

**Table 5.** Model metrics of the final IC _50_ ensemble model.

Metric	Value
Normalized Root Mean Squared Error	0.1228
Root Mean Squared Log Error	0.1336
Normalized Mean Absolute Error	0.1097
Mean Absolute Percentage Error	24.27
Normalized Median Absolute Error	0.1097
Root Mean Squared Error	0.3398
Explained Variance	-1.755
Normalized Root Mean Squared Log Error	0.1379
Median Absolute Error	0.3035
Mean Absolute Error	0.3035

**Table 6.** Top 10 training iterations of the IC _50_ model search, evaluated by Root Mean Squared Error. Note that the top performing model (VotingEnsemble) is the final IC _50_ model discussed in this paper.

Iteration	Preprocessor	Algorithm	Normalized RMSE
498		VotingEnsemble	0.12283293
370	SparseNormalizer	RandomForest	0.132003138
432	StandardScalerWrapper	LightGBM	0.133180215
240	SparseNormalizer	RandomForest	0.133779391
430	StandardScalerWrapper	RandomForest	0.137084337
65	SparseNormalizer	RandomForest	0.13884791
56	SparseNormalizer	RandomForest	0.14417843
68	MaxAbsScaler	ExtremeRandomTrees	0.151925822
470	StandardScalerWrapper	RandomForest	0.152262231
181	MinMaxScaler	LightGBM	0.15279075

## Prediction of resistance status

The second task of this work was to create a machine learning model that can predict the parasite clearance rate (fast versus slow) of malaria isolates. When resistance rates change in a pathogen, it can be indicative of regulatory changes in the pathogen’s genome. These changes can be exploited for the prevention of further resistance spread. Thus, a goal of this work is to understand genes important in the prediction of artemisinin resistance.

### Methods

An *in vivo* transcription data set from Mok *et al.*, (2015) Science ^18^ was used to predict the parasite clearance rate of malaria parasite isolates based on *in vitro* transcriptional profiles (see Table 8).

The training data consists of 1,043 isolates with 4,952 genes from the malaria parasite *Plasmodium falciparum*. For each malaria parasite isolate, transcription data was collected for various *PF3D7* genes. The form of the training dataset contains 1,043 rows and 4,957 columns, as shown in Table 7. The feature to predict is *ClearanceRate*.

**Table 7.** Format of the clearance rate model training data.

Sample_Names	Country	Asexual_ stage hpi_	Kmeans_Grp	PF3D7_ 0100100	…	PF3D7_1480100	ClearanceRate
GSM1427365	Bangladesh	20	B	0.226311	…	-0.64171	Fast
…	…	…	…	…	…	…	…
GSM1427537	Cambodia	12	C	0.81096	…	-1.72825	Slow
…	…	…	…	…	…	…	…
GSM1428407	Vietnam	8	A	0.999095	…	NaN	Fast

**Table 8.** Training dataset information from Mok *et al.*, 2015 ^18^.

	Training Set
Number of isolates	1043
Isolate collection site	Southeast Asia
Isolate collection years	2012–2014
Sample type	*in vivo*
Synchronized?	Not synchronized
Number of samples per isolate	1
Additional attributes	~18 hpi, Non-perturbed, No replicates

### Data preparation

The training data for this use case did not require the same pivoting transformations as in the last use case as each record describes a single isolate. Thus, only the vectorization of the data was necessary, which was performed using the Spark VectorAssembler and then converted into a Numpy-compatible array ^12^. Example code is shown below. Note that this vectorization only kept the numerical columns, which excludes the Country, Kmeans_Grp, and Asexual_stage__hpi_ attributes as they are either absent or contain non-matching factors (i.e. different set of countries) in the testing data.


 1 ## Transform Data using VectorAssembler                                          
 2 assemblerInputs = numericalColumns                                               
 3 assembler = VectorAssembler(inputCols = assemblerInputs, outputCol="features") \ 
 4             .setHandleInvalid("keep")                                            
 5 stages += [assembler]                                                            
 6                                                                                  
 7 prepPipeline = Pipeline().setStages(stages)                                      
 8 pipelineModel = prepPipeline.fit(data)                                           
 9 vectordata = pipelineModel.transform(data)                                       
10                                                                                  
11 ## Convert to Numpy Array                                                        
12 import numpy as np                                                               
13 pddata = vectordata.toPandas()                                                   
14 seriesdata = pddata['features'].apply(lambda x : np.array(x.toArray())) \        
15              .as_matrix().reshape(-1,1)                                          
16 X_train = np.apply_along_axis(lambda x : x[0], 1, seriesdata)                    
17 y_train = pddata['label'].values.reshape(-1,1).ravel()                           
18                                                                                  
19 ## Example Output (X_train) After Vectorization                                  
20 array([[ 0.2263112 , -0.39682897, -1.80458125, ...,        nan,                  
21         -1.30952803, -0.64170958],                                               
22        [ 0.55442743,  0.54200115, -1.56157279, ..., 1.83083869,                  
23          0.21021662, -1.06553341],                                               
24        ...,                                                                      
25        [ 1.24446867, -0.09076431, -1.62156926, ..., 3.18060844,                  
26         -0.43056353,         nan],                                               
27        [ 0.99909549, -1.47208829, -1.91898139, ..., 2.59463935,                  
28         -1.21233458,         nan]])                                              


### Machine learning

Once the 98 individual models were trained, two ensemble models (voting ensemble and stack ensemble) were then created and tested as before. Model search parameters are shown in Table 9.

**Table 9.** Model search parameter settings for the clearance rate model search.

Parameter	Value
Task	Regression
Number of iterations	100
Iteration timeout (minutes)	20
Max cores per iteration	14
Primary metric	weighted area under the receiver operating characteristic curve (AUC)
Preprocess data?	True
k-Fold cross-validations	10 folds

### Results

**Figure 3.**
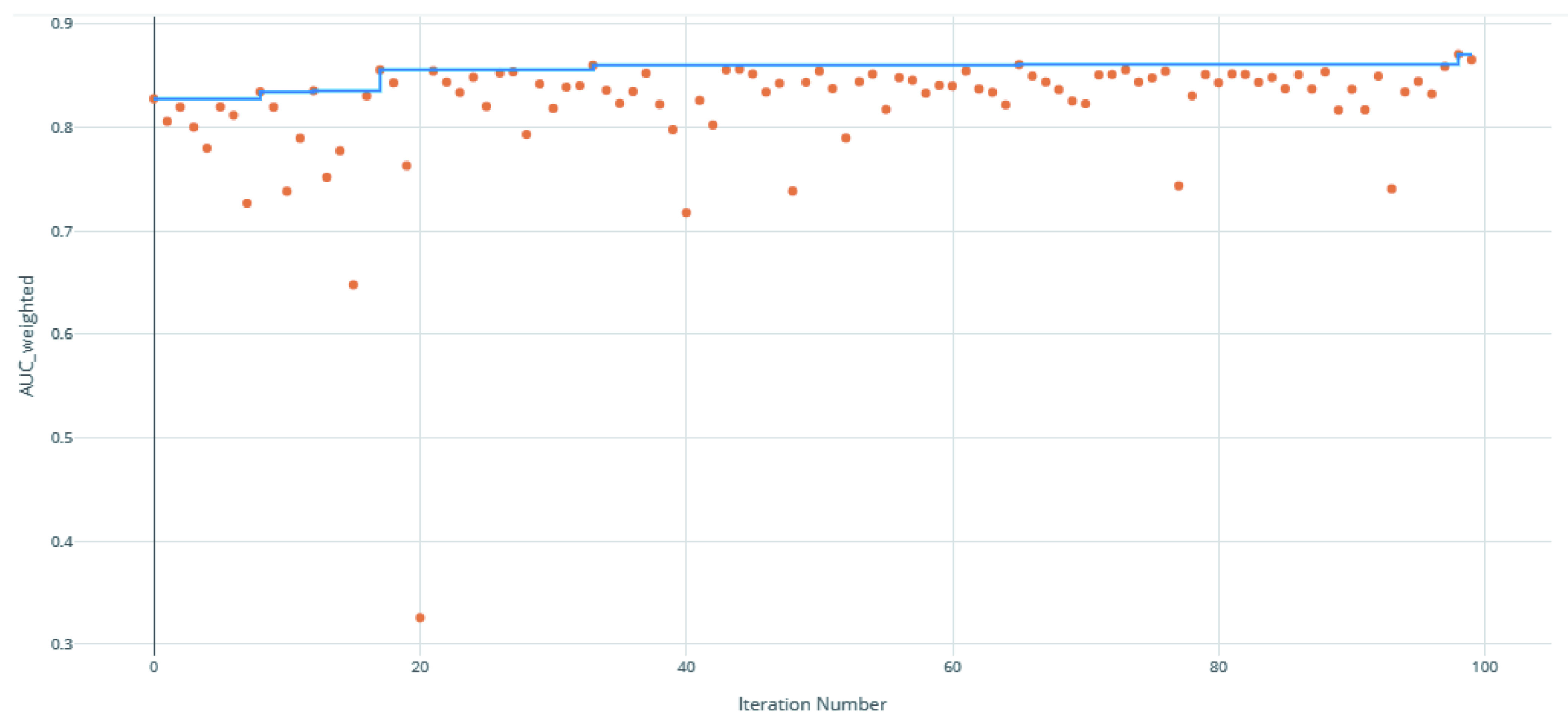
Area under the receiver operating characteristic curve (AUC) by iteration of the clearance rate model. Each orange dot is an iteration with the blue line representing the maximum AUC up to that iteration.

**Figure 4.**
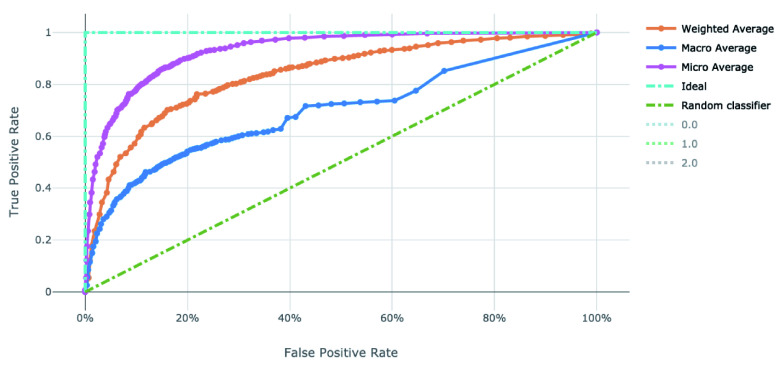
Receiver operating characteristic curve of the clearance rate model.

**Figure 5.**
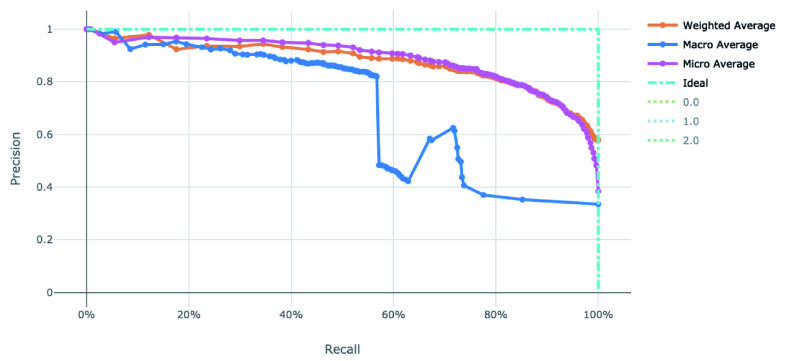
Precision-Recall curve of the clearance rate model.

The voting ensemble model (using soft voting) was selected as the best model, having the highest area under the receiver operating characteristic curve (AUC), as shown in Table 11. The top 10 of the 100 models trained are reported in Table 10. Having a weighted AUC of 0.87 and a weighted F1 score of 0.80, this model is expected to accurately predict isolate clearance rates. A confusion matrix of the predicted results versus actuals is shown in Table 12. See Figure 3 for a visualization of the experiment runs and see Figure 4 and Figure 5 for the ROC and Precision-Recall curves on the best model.

**Table 10.** Top 10 training iterations of the clearance rate model search. Note that the top performing model (VotingEnsemble) is the clearance rate model discussed in this paper.

Iteration	Preprocessor	Algorithm	Weighted AUC
98		VotingEnsemble	0.870471056
99		StackEnsemble	0.865215516
65	StandardScalerWrapper	LogisticRegression	0.86062304
33	StandardScalerWrapper	LogisticRegression	0.859881677
97	StandardScalerWrapper	LogisticRegression	0.858791006
44	StandardScalerWrapper	LogisticRegression	0.856105491
73	StandardScalerWrapper	LogisticRegression	0.855502817
17	RobustScaler	SVM	0.855452622
43	StandardScalerWrapper	LogisticRegression	0.855368394
61	RobustScaler	LogisticRegression	0.854357599

**Table 11.** Model metrics of the final clearance rate ensemble model.

Metric	Accuracy
f1_score_macro	0.6084
AUC_micro	0.9445
AUC_macro	0.8475
recall_score_micro	0.8101
recall_score_weighted	0.8101
average_precision_score_weighted	0.8707
weighted_accuracy	0.8585
precision_score_macro	0.6217
precision_score_micro	0.8101
balanced_accuracy	0.6027
log_loss	0.4455
recall_score_macro	0.6027
precision_score_weighted	0.8
AUC_weighted	0.8705
average_precision_score_micro	0.8911
f1_score_weighted	0.8019
f1_score_micro	0.8101
norm_macro_recall	0.354
average_precision_score_macro	0.7344
accuracy	0.8101

**Table 12.** Confusion matrix of clearance rate predictions versus actual.

Class		Prediction		
		Fast (ID: 0)	Slow (ID: 1)	Null (ID: 2)
Actual	**Fast (ID: 0)**	661	74	0
	**Slow (ID: 1)**	115	184	0
	**Null (ID: 2)**	6	3	0

**Figure 6.**
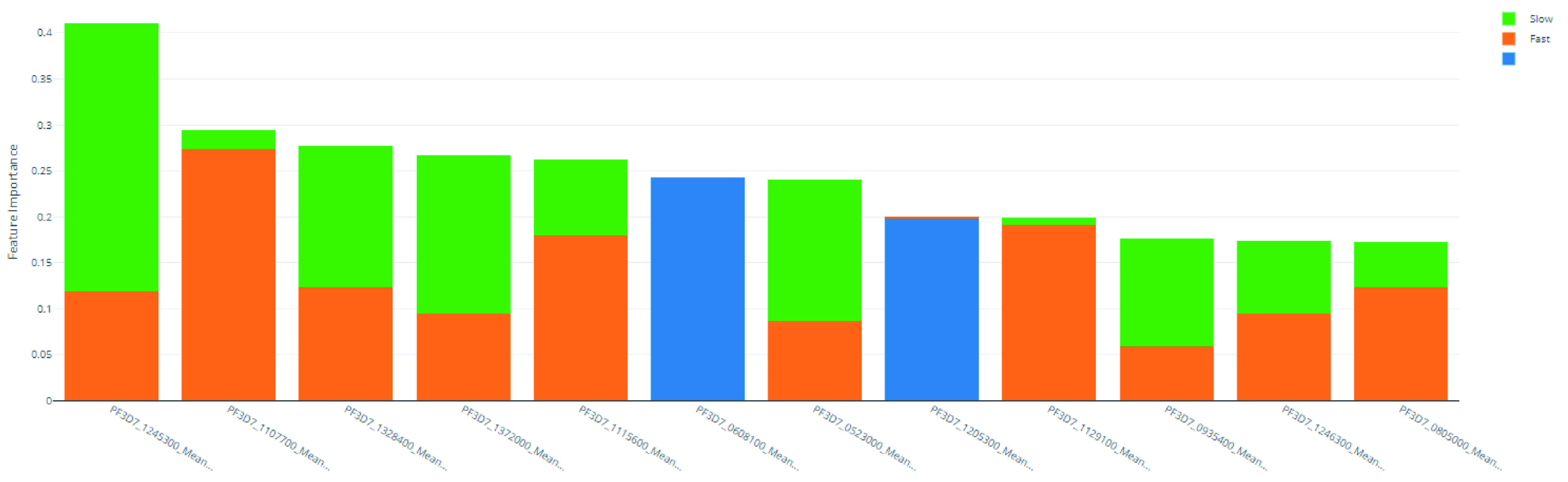
Derived feature importances using the black box mimic model explanation of the clearance rate model.

subsectionFeature importance Feature importances were calculated using mimic-based model explanation of the ensemble model ^19^. The mimic explainer works by training global surrogate models to mimic blackbox models. The surrogate model is an interpretable model, trained to approximate the predictions of a black box model as accurately as possible. See Figure 6 and Table 13.

**Table 13.** Top 10 PF3D7 genes (features) in predicting clearance rate.

Rank	PF3D7 Gene	"Slow" Importance	"Fast" Importance	"NULL" Importance	Overall Importance
1	PF3D7_1245300	0.292	0.118	0.000	0.410
2	PF3D7_1107700	0.020	0.274	0.000	0.294
3	PF3D7_1328400	0.154	0.123	0.000	0.277
4	PF3D7_1372000	0.172	0.095	0.000	0.267
5	PF3D7_1115600	0.083	0.179	0.000	0.262
6	PF3D7_0608100	0.000	0.000	0.243	0.243
7	PF3D7_0523000	0.154	0.087	0.000	0.241
8	PF3D7_1205300	0.000	0.002	0.197	0.199
9	PF3D7_1129100	0.008	0.191	0.000	0.199
10	PF3D7_0935400	0.117	0.058	0.000	0.175

**Table 14.** Scaling function information for machine learning model search ^20^.

Scaling and Normalization	Description
StandardScaleWrapper	Standardize features by removing the mean and scaling to unit variance
MinMaxScalar	Transforms features by scaling each feature by that column’s minimum and maximum
MaxAbsScaler	Scale each feature by its maximum absolute value
RobustScalar	This Scaler features by their quantile range
PCA	Linear dimensionality reduction using singular value decomposition of the data to project it to a lower dimensional space
TruncatedSVDWrapper	This transformer performs linear dimensionality reduction by means of truncated singular value decomposition. Contrary to PCA, this estimator does not center the data before computing the singular value decomposition. This means it can efficiently work with sparse matrices.
SparseNormalizer	Each sample (each record of the data) with at least one non-zero component is re- scaled independently of other samples so that its norm (L1 or L2) equals one

## Discussion

By using distributed processing of the data preparation, we can successfully shape and manage large malaria datasets. We efficiently transformed a matrix of over 40,000 genetic attributes for the IC _50_ use case and over 4,000 genetic attributes for the resistance rate use case. This was completed with scalable vectorization of the training data, which allowed for many machine learning models to be generated. By tracking the individual performance results of each machine learning model, we can determine which model is most useful. In addition, ensemble modeling of the various singular models proved effective for both tasks in this work.

The resulting model performance of both the IC _50_ model and the clearance rate model show relatively adequate fitting of the data for their respective predictions. While additional model tuning may provide a lift in model performance, we have demonstrated the utility of ensemble modeling in these predictive use cases in malaria.

In addition, this exercise helps to quantify the importance of genetic features, spotlighting potential genes that are significant in artemisinin resistance. The utility of these models will help in directing development of alternative treatment or coordination of combination therapies in resistant infections.

## Preprint

An earlier version of this article can be found on bioRxiv (doi: 10.1101/856922).

## Data availability

### Underlying data

The challenge datasets are available from Synapse ( https://www.synapse.org/; Synapse ID: syn18089524). Access to the data requires registration and agreement to the conditions for use at: https://www.synapse.org/#!Synapse: syn18089524.

Challenge documentation, including the detailed description of the Challenge design, data description, and overall results can be found at: https://www.synapse.org/#!Synapse:syn16924919/wiki/583955.

Whole genome expression profiling of artemsinin-resistant Plasmodium falciparum field isolates, Accession number GSE59099: https://www.ncbi.nlm.nih.gov/geo/query/acc.cgi?acc=GSE59099.

## Software availability

- Source code available from: https://github.com/colbyford/malaria_DREAM2019
- Archived source code at time of publication: https://doi.org/10.5281/zenodo.3590459 ^21^
- License: GPL-3.0
