# Supplementary material for: Ensemble machine learning modeling for the prediction of artemisinin resistance in malaria
Source: F1000Res. 2020 Jun 25;9:62. Originally published 2020 Jan 29. [Version 5] doi: 10.12688/f1000research.21539.5 (PMC9274019; doi:10.12688/f1000research.21539.5)
Supplement: Peer review history

# Peer review: Reviewer response for version 5

**DOI:** 10.5256/f1000research.27621.r139927

**Published:** 2022-07-11

**Authors:** Alyssa E Barry, Myo Naung

**Affiliations:** 1 IMPACT, School of Medicine, Deakin University and Burnet Institute, Melbourne, Victoria, Australia; 2 Walter and Eliza Hall Institute, University of Melbourne, Melbourne, Australia

This is commendable work by the authors making use of two publicly available datasets – the 2019 DREAM Malaria Challenge and an *in vivo* transcription data set from Mok *et al.,* (2015) to create a confident machine learning model predicting IC _50_ (the rate at which parasites respond to artmesinin) and the transcriptional features (gene expression) involved in fast vs slow parasite clearance rates. Source codes were also made available to public for reproducibility. The manuscript would benefit from more structure in the “ **methods**” and “ **results” **sections to present clearer analysis workflow and results. Adding more explanation and discussion of biological significance from the generated models should improve the quality of the manuscript. There are a few specific suggestions for the authors in a revision of their manuscript below.

Major

 The strongest suggestion is to re-structure the methods section. Rather than having separate sections (“method”, “data preparation”, “results”) for each machine learning exercise, I suggest merging some of these paragraphs. For instance, the entire method section describing model generation (page 3-5) to predict IC _50_ can be possibly renamed as “machine learning method to predict IC _50_”. Along this line, the paragraph titled “Prediction of artemisinin IC _50_” should merge with this section. A similar arrangement is also suggested for paragraphs from page 5-7. Adding a generic workflow figure should clarify some of these issues. Both of the results sections (page 5 & 8) should come after methods. 

 Authors used datasets specific to *Plasmodium falciparum* malaria and thus title should reflect about *P. falciparum* malaria. The results section paragraph (page 8) should be expanded to include more explanation of results from figure-6, which can be connected to the previously known attributes of these top-30 hits as discussed in the 4 ^th^ paragraph of discussion (page 13).

Minor

- Abstract 2 ^nd^ paragraph– indicate ‘2019 DREAM Malaria Challenge’
- Methods typo -  “This yields a total of at eight data points for each isolate."
- Description of table-3 (page 4) such as number of rows, columns in training data can be moved to the Github page. The same suggestions for table-1 and table-7.
- A simple definition or explanation of “Caruana ensemble” selection algorithm (page 5) should be added.
- Mean Absolute Percentage Error (MAPE) of algorithms should be added to table-6 (page 6) because it is mentioned in the results section (page 5) that the voting ensemble model has chosen as the best model based on RMSE and MAPE.
- Paragraph starting with “Note that the ....” (page 8) should be considered as figure legends for figure 4 and 5.
- Paragraph explaining the use of mimic explainer over PCA (page 10) should be considered to move to discussion possibility merging with 4 ^th^

Is the rationale for developing the new method (or application) clearly explained?

Yes

Is the description of the method technically sound?

Yes

Are the conclusions about the method and its performance adequately supported by the findings presented in the article?

Partly

If any results are presented, are all the source data underlying the results available to ensure full reproducibility?

Yes

Are sufficient details provided to allow replication of the method development and its use by others?

Partly

Reviewer Expertise:

malaria, genomics, computational biology

We confirm that we have read this submission and believe that we have an appropriate level of expertise to confirm that it is of an acceptable scientific standard, however we have significant reservations, as outlined above.

# Peer review: Reviewer response for version 5

**DOI:** 10.5256/f1000research.27621.r65540

**Published:** 2020-07-10

**Authors:** Sameer K. Antani

**Affiliations:** 1 Communications Engineering Branch, National Library of Medicine, National Institutes of Health, Bethesda, MD, USA

The authors have updated the article but there is limited update on machine learning elements, or it is not apparent from the web-based interface. I am willing to accept the article related to prior comments, and also recognizing that the work is limited by the data from the DREAM challenge.

Is the rationale for developing the new method (or application) clearly explained?

Partly

Is the description of the method technically sound?

Partly

Are the conclusions about the method and its performance adequately supported by the findings presented in the article?

Partly

If any results are presented, are all the source data underlying the results available to ensure full reproducibility?

Yes

Are sufficient details provided to allow replication of the method development and its use by others?

Partly

Reviewer Expertise:

NA

I confirm that I have read this submission and believe that I have an appropriate level of expertise to confirm that it is of an acceptable scientific standard.

# Peer review: Reviewer response for version 4

**DOI:** 10.5256/f1000research.26770.r63887

**Published:** 2020-06-22

**Authors:** Jeremy Burrows

**Affiliations:** 1 Medicines for Malaria Venture (MMV), Geneva, Switzerland

Page 3: Artemisinin-based therapies are described as being among the best treatment options for falciparum malaria. ACTs are the mainstay therapy and are, definitively, the best treatment options. This should be altered.

 Page 3: The underlying biology of artemisinin partial resistance is becoming clearer – the authors should cite Kelch13-defined endocytosis pathway mediates artemisinin resistance in malaria parasites ^1^.

 Page 3: In terms of predicting the IC50 of DHA on parasites, the authors really need to comment on the importance of time point and whether the parasites are synchronous or asynchronous. Usually growth inhibition assays are 48h-72h with asynchronous parasites and these result in virtually no differences in the IC50s between WT and highly resistant K13 mutant strains – indeed, this is why artemisinin partial resistance took so long to be identified. Table 1 does show the timepoint (which is good) but the synchronicity of the isolate is not mentioned. Also did the group include well characterized control lab-adapted strains (both resistant and WT)? What is the range of IC50s in the data set?

 Table 1 – Abbreviations need to be described. I know what DHA is, but some readers may not. What is UT?

 The computational discussion is beyond me, but I was trying to work out exactly what the authors were claiming. Is the conclusion of the first step that the IC50 can be predicted based on the transcriptomic analysis, given full genomic information of an isolate? If so, that could be interesting in predicting phenotype from genotype (in the absence of phenotypic data), but if, on the other hand, it simply confirms resistance will be evident when certain mutations are involved, then that is not so helpful as we know that already. Can the authors very clearly, in layman’s terms, explain what value their model offers to the parasitology community? The same points relate to parasite clearance rate? If the model is simply telling us what we know already then that is significantly less useful or interesting than if it predicts things that we do not yet know. Some very clear explanations of the hypotheses and conclusions are needed for non-computational parasitologists to understand the merit of this work. This is not approvable without such clarity, on the assumption that other reviewers with computational expertise have approved the underlying methods.

 The identification of PF3D71245300, a NEDD8-conjugating enzyme UBC12 and PF3D71107700 seem to me to be predicted genes for slow and fast clearance are the main conclusions from this work and there should be a stronger statement with respect to the need for follow-up biology to confirm these.

 I would like to have seen a plot of predicted vs actual IC50 and predicted vs actual clearance rate in a form that is easily interpretable (perhaps it’s there for those in the ‘know’). I was still left unclear as to how good the models were; the authors described them as ‘adequate’ which sounds rather underwhelming.

 In short – the work may have merit, but it is not communicated in a form that makes it clear what the added value is to use the model and what the actual quality and impact is of the model.

Is the rationale for developing the new method (or application) clearly explained?

No

Is the description of the method technically sound?

Partly

Are the conclusions about the method and its performance adequately supported by the findings presented in the article?

Partly

If any results are presented, are all the source data underlying the results available to ensure full reproducibility?

Partly

Are sufficient details provided to allow replication of the method development and its use by others?

Partly

Reviewer Expertise:

Drug discovery, malaria, parasitology (not computational modelling).

I confirm that I have read this submission and believe that I have an appropriate level of expertise to confirm that it is of an acceptable scientific standard, however I have significant reservations, as outlined above.

## References

[1] : A Kelch13-defined endocytosis pathway mediates artemisinin resistance in malaria parasites. *Science* .2020;367(6473) :51-59 3189671010.1126/science.aax4735

# Response

**Published:** 2020-06-22

**Authors:** Colby Ford

**Affiliations:** University of North Carolina at Charlotte, USA

Thank you for your review. We have added the additional context about ACTs, and the *kelch13 *gene from the Birnbaum paper. In addition, we have included information about how this work is of merit and applicable to the broader field of parasitology. We also included information in the discussion about the need for biological ( *in vitro*) validation of these findings, but that this work helps to "bubble up" the most probable/important things to test first.

 As for the specific questions about the data used in this study, we are still waiting on the overall DREAM Challenge write up and release to occur, which should contain must more in-depth information about the lab procedures (timepoints, test, etc.) and data collection. We are open to adding this information into our paper as well once we can get it from the DREAM Challenge.

 Though only the ROC Curves are shown in the paper, all of the figures for model performance are in the GitHub repository. The plot of actual vs. predictive performance (a.k.a. calibration curve) is here: https://github.com/colbyford/malaria_DREAM2019/blob/master/SubChallenge2/model/amls_model_7-31-19/Calibration.PNG

# Peer review: Reviewer response for version 3

**DOI:** 10.5256/f1000research.25874.r62868

**Published:** 2020-05-18

**Authors:** Sameer K. Antani, Stefan Jaeger

**Affiliations:** 1 National Library of Medicine, National Institutes of Health, Bethesda, USA; 2 Communications Engineering Branch, National Library of Medicine, National Institutes of Health, Bethesda, MD, USA

The authors have addressed several, but not all of the reviewers’ comments. The description of the state-of-the-art could be stronger. For example, the authors should discuss the status quo in machine learning for malaria drug-resistance detection, and the status/results of the DREAM Competition in particular, including the context of the data used. Some questions remain regarding the computation of averages in the ROC and precision-recall curves in Figures 4 and 5, see for example the bump in the latter (blue curve). The authors have not explained Figure 6, as reviewers asked them to do (labeling and legend fonts are too small). The authors also don’t explain how they do the testing (size of test set, evaluation scheme etc.) I am not sure about the usefulness of Figure 2 - Sensitivity and specificity are more intuitive measures than squared errors.

Is the rationale for developing the new method (or application) clearly explained?

Partly

Is the description of the method technically sound?

Partly

Are the conclusions about the method and its performance adequately supported by the findings presented in the article?

Partly

If any results are presented, are all the source data underlying the results available to ensure full reproducibility?

Yes

Are sufficient details provided to allow replication of the method development and its use by others?

Partly

Reviewer Expertise:

machine learning, artificial intelligence, data science, malaria screening

We confirm that we have read this submission and believe that we have an appropriate level of expertise to state that we do not consider it to be of an acceptable scientific standard, for reasons outlined above.

# Response

**Published:** 2020-05-19

**Authors:** Colby Ford

**Affiliations:** University of North Carolina at Charlotte, USA

We appreciate the reviewer's comments and have made some updates to the manuscript to reflect some figure quality issues and to address some points of confusion.

 In this revision, we have addressed the reviewer's comments around the precision-recall curve and the ROC curve by better explaining the variation in the P-R curve and defining the micro, macro, and weighted average metrics shown in the figures. In addition, we have replaced the feature importance bar chart (Figure 6) with a higher quality version, which should be much more readable. We have also better described the model evaluation process.

**Note: **This article is part of a larger DREAM Challenge, from which a larger compilation manuscript will be written at a later date. As such, we cannot yet publish the data used in this work. We also cannot control the data used in this work as we were to use the data provided by the 2019 Malaria DREAM Challenge. Thus, we should not be evaluated on the lack of data, lack of a public testing dataset (which will be published in the future parent article), lack of a public evaluation scheme, the status/results of the DREAM Competition, or the full context of the data used.

# Peer review: Reviewer response for version 2

**DOI:** 10.5256/f1000research.24636.r60584

**Published:** 2020-03-17

**Authors:** Sameer K. Antani, Stefan Jaeger

**Affiliations:** 1 National Library of Medicine, National Institutes of Health, Bethesda, USA; 2 Communications Engineering Branch, National Library of Medicine, National Institutes of Health, Bethesda, MD, USA

The authors present a machine learning approach for detecting malaria drug-resistance based on genetic attributes. To this end, they train many different models, which they combine with known ensemble methods like voting. The detection of malaria drug resistance is an important medical problem and the application of machine learning in this context deserves further exploration. However, the paper has several shortcomings that the authors need to address:

- The author should provide a better description of the state-of-the-art and existing literature at the beginning of their paper. 
- Also demonstrate the need for such an approach. It is implicitly suggested, but greater clarity is needed on what gaps this approach fills. This can be addressed through previous bullet also.
- The overall structure of the paper lacks clarity and concrete results. The authors claim that their exercise helps to “quantify the importance of genetic features, spotlighting potential genes that are significant in artemisinin resistance. The utility of these models will help in directing development of alternative treatment or coordination of combination therapies in resistant infections.” However, the experimental validation of these statements is insufficient, and the derived feature importance need to be discussed in more detail to convince the reader. In this context, Figure 6 need to be explained and discussed. What is the black block mimic model? Why has it been chosen by the authors for ranking features? In what way do other feature ranking schemes like PCA differ? 
- The paper describes two experiments: a regression experiment with the IC50 value as target, and a classification experiment with three different parasite clearance rates. However, both experiments need further justification. In the first experiment, the number of rows (patterns) seems to be very small compared to the number of features (genetic attributes), which makes over-training very likely. The authors need to comment on this and address the issue if possible. In the second experiment, it is unclear how the three different clearance rates relate to drug-resistance. What is the correlation between these classes and drug-resistance? Why have the authors trained many more models for the first experiment?
- Listing of source code for formatting data is unnecessary and not suitable for a research paper. They have provided links to their code so including it in the paper seems superfluous, unless they want to make a point about it, which is absent. Further, that their example output after vectorization contains NaNs does not inspire confidence in the quality of the code; and, obviously needs further discussion.
- The authors also cite that an earlier version of this article is available on bioRxiv. They should include discussion on what improvements are in this work that substantially improve over that.

Is the rationale for developing the new method (or application) clearly explained?

Partly

Is the description of the method technically sound?

Partly

Are the conclusions about the method and its performance adequately supported by the findings presented in the article?

Partly

If any results are presented, are all the source data underlying the results available to ensure full reproducibility?

Yes

Are sufficient details provided to allow replication of the method development and its use by others?

Partly

Reviewer Expertise:

machine learning, artificial intelligence, data science, malaria screening

We confirm that we have read this submission and believe that we have an appropriate level of expertise to state that we do not consider it to be of an acceptable scientific standard, for reasons outlined above.

# Response

**Published:** 2020-04-09

**Authors:** Colby Ford

**Affiliations:** University of North Carolina at Charlotte, USA

We sincerely appreciate the reviewers' feedback on this work and have improved the article based on your recommendations.

 We have addressed each comment as follows in the article:

- Added additional examples of ML-based work in genomics, other tropical diseases, and in malaria.
- Added a brief explanation about the utility of this approach and its benefit over manual analysis.
- Addressed the reviewers' questions in the article around the explanability and black box methods and gave examples of the role of certain important genes identified here.
- Addressed the small observation size and the training of many models in the article and have better explained the relationship between drug resistance and parasite clearance rates. Further information on this data can be found in Mok et al., 2015. Also, the reason the second use case has fewer models trained was due to performance and risk of overfitting. However, with more observations, the machine learning modeling performance may increase with additional training and tuning time.
- The example code segments have been removed from the article.
- For the data quality concern, this is the data provided by the DREAM competition, thus isn't something we can control.
- The previous version on bioRxiv is nearly identical and was published there until the gateway was set up on F1000 and the publication embargo was lifted.
